# Relative Importance of Aortic Stiffness and Volume as Predictors of Treatment-Induced Improvement in Left Ventricular Mass Index in Dialysis

**DOI:** 10.1371/journal.pone.0135457

**Published:** 2015-09-10

**Authors:** Panagiotis I. Georgianos, Rajiv Agarwal

**Affiliations:** Department of Medicine, Indiana University School of Medicine and Richard L. Roudebush Veterans Administration Medical Center, Indianapolis, IN, United States of America; Scuola Superiore Sant'Anna, ITALY

## Abstract

This study aimed to explore the relative contribution of aortic stiffness and volume in treatment-induced change of left ventricular mass in dialysis. Hypertension in Hemodialysis Patients Treated with Atenolol or Lisinopril trial compared the effect of lisinopril versus atenolol in reducing left ventricular mass index; 179 patients with echo measurements of aortic pulse wave velocity and left ventricular mass at baseline were included. In unadjusted analysis, overall reductions of 26.24 g/m^2^ (95% CI: -49.20, -3.29) and 35.67 g/m^2^ (95% CI: -63.70, -7.64) in left ventricular mass index were noted from baseline to 6 and 12 months respectively. Volume control emerged as an important determinant of regression of left ventricular mass index due to the following reasons: (i) additional control for change in ambulatory systolic blood pressure mitigated the reduction in left ventricular mass index in the statistical model above [6-month visit: -18.6 g/m^2^ (95% CI: -43.7, 6.5); 12-month visit: -22.1 g/m^2^ (95% CI: -52.2, 8.0)] (ii) regression of left ventricular hypertrophy was primarily due to reduction in left ventricular chamber and not wall thickness and (iii) adjustment for inferior vena cava diameter (as a proxy for volume) removed the effect of time on left ventricular mass index reduction [6-month visit: -6.6 g/m^2^ (95% CI: (-41.6, 28.4); 12-month visit: 0.6 g/m^2^ (95% CI: -39.5, 40.7)]. In contrast, aortic pulse wave velocity was neither a determinant of baseline left ventricular mass index nor predictor of its reduction. Among dialysis patients, ambulatory systolic pressure, a proxy for volume expansion, but not aortic stiffness is more important predictor of reduction in left ventricular mass index. Improving blood pressure control via adequate volume management appears as an effective strategy to improve left ventricular hypertrophy in dialysis.

## Introduction

Left ventricular hypertrophy (LVH) is an independent and powerful predictor of cardiovascular morbidity and mortality both in the general population [1] and among patients with chronic kidney disease (CKD) [2], including those receiving maintenance hemodialysis therapy [3, 4]. Development of LVH is an early event in the natural course of CKD; LVH progresses over time in parallel with deterioration of renal function [2]. Thus, in the vast majority of patients who reach end-stage renal disease (ESRD) and initiate renal replacement therapy LVH is already established. Among chronic hemodialysis patients it remains largely unclear which factors (left ventricular cavity dimension or ventricular wall thickness) determine LVH change over time [5].

Among long-term hemodialysis patients, LVH can be either due to hypertrophy of the left ventricular (LV) wall or dilatation of the LV chamber. Particularly in the setting of volume expansion, LVH appears to be due to left ventricular chamber dilatation, especially when patients are unable to reach “dry weight” [5]. In a previous randomized trial, we have shown that among hypertensive hemodialysis patients, elevated left ventricular mass index (LVMI) is a marker reflecting volume excess and probing of dry weight during dialysis is associated with short-term improvement in LVH [6]. This is mainly due to reduction in the LV chamber diameter rather than regression of LV wall hypertrophy.

Another factor proposed to play an important role in promoting the long-term progression of LVH in hemodialysis patients is impaired mechanical properties of the aorta and large conduit arteries due to accelerated arterial stiffening [7, 8]. Arteriosclerosis is considered as one of the main determinants of increased aortic systolic pressure and pulse pressure, leading to augmented LV work load. Although cross-sectional studies have supported the notion that aortic stiffness and LVMI maybe interrelated [9, 10], the role of aortic stiffness as predictor of longitudinal change of LVH has never been previously investigated among hemodialysis patients.

The Hypertension in Hemodialysis treated with Atenolol or Lisinopril (HDPAL) study compared the effect of atenolol versus lisinopril in causing regression of LVH in hemodialysis patients [11]. In this trial, we directly measured arterial stiffness and volume markers. Accordingly, the aim of the present analysis was to investigate among hypertensive hemodialysis patients with echocardiographic LVH the relative importance of aortic stiffness and volume as predictors of treatment-induced decline in LVMI.

## Materials and Methods

### Study design

The design of the HDPAL randomized trial was previously published [11]. In brief, HDPAL study compared the effect of 12-month treatment with atenolol versus lisinopril on causing regression in LVMI in a cohort of 200 ESRD patients receiving standard thrice-weekly hemodialysis therapy for at least 3 months. All patients had hypertension confirmed by 44-hour interdialytic ambulatory blood pressure monitoring (ABPM) and echocardiographic LVH. Patients were excluded from the study in case of: (i) chronic atrial fibrillation; (ii) body mass index (BMI) ≥40 kg/m^2^; (iii) history of missing one or more dialysis treatments within the previous month; (iv) severe chronic obstructive pulmonary disease; (v) stroke or myocardial infarction during the previous 6 months; (vi) known contraindication to the randomized drugs.

After a 3-week run-in washout period, eligible patients were randomly assigned in a 1:1 ratio to receive open-label atenolol 25 mg or lisinopril 10 mg both administered 3 times per week post dialysis. The protocol of the study prespecified dose up-titration of randomized drugs, administration of other antihypertensive agents on top to the initial regimen, adequate volume management during dialysis and reducing sodium concentration in the dialysate, aiming to lower monthly recorded home blood pressure (BP) below the target of 140/90 mmHg. In case of uncontrolled home BP during the monthly follow-up visits, felodipine or amlodipine 10mg once daily was added, followed by other antihypertensive medications in the following order: doxazosin, minoxidil and guanfacine.

### Ethics statement

All protocol procedures were conducted in accordance with the Declaration of Helsinki (2000 Amendment) and informed written consent was obtained from each patient prior to study enrollment. Study protocol was approved by the Institutional Review Board of Indiana University and the Research and Development Committee of the Roudebush VA Medical Center, Indianapolis, IN, USA.

### Study evaluations

#### Left ventricular mass index

Two-dimensional guided M-mode echocardiograms were performed by dedicated technicians shortly after dialysis procedure with the use of a digital cardiac ultrasound device (Cy-press Acuson, Siemens Medical). Echocardiograms were performed shortly after dialysis, since this period permits better control of hydration status and is associated with the least intravascular volume. The protocol specified the recording of at least 6 cycles of 2-dimensional parasternal long- and short-axis left ventricular views with optimal orientation of the cursor beam to derive additional M-mode recordings [12]. Each patient underwent 6 M-mode measurements of interventricular septal thickness (IVST), LV posterior wall thickness (LVPWT) and left ventricular internal diameter (LVID) all in end-diastole, according to standard guidelines of the American Society of Echocardiography. Left ventricular mass was calculated on the basis of a previously validated formula: LV mass (g) = 0.832 x [(IVST+LVID+LVPWT)3 - (LVID)^3^]+0.60 [12]. LV mass ≥104 g/m^2^ in females and ≥116 g/m^2^ in males were considered diagnostic of LVH. Echocardiographic evaluations were performed at baseline and were repeated at 6 and 12 months of follow-up.

#### Inferior vena cava measurement

Inferior vena cava (IVC) was imaged at the level just below the diaphragm in the hepatic segment by 2-dismentional guided, M-mode echocardiography over 6 cardiac cycles. IVC diameter was measured just before the P wave of the electrocardiogram during end expiration and end inspiration, while avoiding Valsalva-like maneuvers. Collapse index was defined as (maximal diameter on expiration—minimal diameter on deep inspiration)/maximal diameter on expiration X 100. Like LV mass, IVC diameter was indexed for body surface area. IVC diameter has previously been validated among long-term hemodialysis patients as a proxy for volume status [13].

#### Aortic stiffness

Arterial stiffness was assessed by measuring aortic pulse wave velocity (PWV) through direct visualization of the descending aorta with the use of the above mentioned echo-Doppler technique (Acuson Cypress, Seimens Medical). Flow pulse was recorded by continuous Doppler from the root of the left subclavian artery and just proximal to the bifurcation of the abdominal aorta with simultaneous electrocardiographic recording [14]. Length of the descending aorta was estimated by measuring the body surface distance from the suprasternal notch to the recording site of aortic signal (near umbilicus). Time elapsed from the peak of the R wave to the foot of the systolic impulse was recorded over six beats. The length of the descending aorta divided by the difference between transit times was calculated to yield aortic PWV [14].

#### Ambulatory BP monitoring

ABPM was started immediately after the mid-week dialysis session and terminated immediately prior to the subsequent dialysis, covering a whole 44 hour interdialytic period. A cuff of appropriate size was fitted to the non-access arm and ambulatory BP recordings were obtained every 20 min during the day-time period (6 AM to 10 PM) and every 30 min during night-time period (10 PM to 6 AM) with a Spacelab 90207 monitor (SpaceLabs Medical, Redmond, WA). Hourly means were calculated. These means were thereafter averaged over the entire interdialytic period to provide mean 44-hour ambulatory systolic BP (SBP) and diastolic BP (DBP). Following the current international recommendation for ABPM, all patients were given instructions to maintain their usual daily activities during the recording period [15].

### Outcome and predictor variables

LVMI, LVID, IVST, LVPWT at baseline evaluation and their longitudinal change with therapy were the outcome variables of this analysis. The primary predictor variables were baseline aortic PWV, study visits and their interaction terms as explained in detail below. We further adjusted the above model for interdialytic ambulatory SBP over 44 hours as a proxy for dry-weight. Also, we adjusted the model separately for IVC diameter as a proxy for volume status.

### Statistical analysis

Continuous variables were expressed as mean ± standard deviations (mean±SDs) and categorical variables as absolutes frequencies and percentages. We divided the study population into tertiles according to the level of baseline aortic PWV. Comparison of demographic, clinical and echocardiographic parameters among PWV tertiles was performed with regression analysis for continuous data and with chi-square test for categorical data.

The primary statistical method used was mixed linear model analysis with fixed and random effects. Maximal likelihood estimates were used to estimate marginal means. The outcome variable was LVMI. To explore the association of baseline aortic PWV with baseline and treatment-induced reduction in LVMI at 6 and 12 months, we used visits (at 0, 6, and 12 months) as indicator variables. The independent fixed predictors were visit (as indicator variable), aortic PWV (as continuous variable), and the interaction of these two terms. Random intercept component was the patient and random slopes the visits. LVID, IVST and LVPWT were also analyzed using separate mixed effects linear models.

The initial models were subsequently adjusted for the following factors: age, gender, race (black or non-black), smoking, diabetic status, hemoglobin, history of pre-existing cardiovascular disease (defined as myocardial infarction, coronary revascularization, hospitalized congestive heart failure, peripheral vascular disease and ischemic stroke), treatment arm (atenolol or lisinopril) and the interaction term of the randomized drug with follow-up visits.

Subsequently, in order to investigate whether treatment-induced regression in LVH is explained by BP reduction, we further adjusted the models for the mean interdialytic ambulatory SBP at baseline and its change throughout the trial.

Finally, in order to investigate whether treatment-induced regression in LVH is explained by volume reduction, we further adjusted the models for the post dialysis IVC diameter adjusted for the body surface area. These measurements were carried out with each measurement of LVMI.

Statistical analysis was performed with Stata version 11.2 (Stata Corp., College Station, TX, USA). A two-sided p value of <0.05 was considered statistically significant.

## Results

### Baseline characteristics of study participants

From a total of 200 hypertensive hemodialysis patients with echocardiographic LVH enrolled in the HDPAL study between August 2005 and September 2013, valid echocardiographic measurements of LVMI and aortic PWV were available in 179 subjects. Of the original 200 participants, 21 were excluded from the present analysis due to the following reasons: 17 subjects did not have their PWV assessed and 4 subjects had technically inaccurate PWV measurement at study enrollment. Among 179 HDPAL participants included in this analysis, 114 patients were assessed at the 6-month and 83 patients were assessed at the 12-month follow-up visit.

Baseline characteristics of study participants according to the tertile of baseline aortic PWV are presented in Table 1. The study included 119 male and 60 female hemodialysis patients with a mean age of 52.1±12.4 years who were predominantly black in race (87.2%). Patients lying within the highest PWV tertile were older in age and had a greater prevalence of diabetes mellitus. However, gender, race, history of previous cardiovascular disease, smoking, type of dialysis access and other clinical and laboratory evaluations were evenly distributed among PWV subgroups.

**Table 1:** Baseline characteristics of study participants according to the tertile of baseline aortic pulse wave velocity.

Clinical characteristic	PWV tertile Low	PWV tertile Medium	PWV tertile High	Overall	P Value
N	60	60	59	179	
Pulse wave velocity (m/s)	5 ± 0.8	7.2 ± 0.6	10.6 ± 2.2	7.6 ± 2.7	<0.001
Age (y)	47.7 ± 10.9	51.1 ± 12	57.7 ± 12.4	52.1 ± 12.4	<0.001
Male sex n(%)	38 (63.3%)	41 (68.3%)	40 (67.8%)	119 (66.5%)	0.82
Blacks n(%)	54 (90%)	53 (88.3%)	49 (83.1%)	156 (87.2%)	0.5
Hispanic n(%)	0 (0%)	1 (1.7%)	0 (0%)	1 (0.6%)	0.37
Atenolol n(%)	28 (46.7%)	33 (55%)	28 (47.5%)	89 (49.7%)	0.6
Diabetes mellitus n(%)	14 (23.3%)	26 (43.3%)	35 (59.3%)	75 (41.9%)	<0.001
Anuric n(%)	37 (60.7%)	40 (65.6%)	42 (73.7%)	119 (66.5%)	0.14
Hospitalized heart failure n(%)	15 (25%)	21 (35%)	16 (27.1%)	52 (29.1%)	0.45
Coronary artery disease n(%)	13 (21.7%)	18 (30%)	13 (22%)	44 (24.6%)	0.49
Coronary revascularization n(%)	1 (1.7%)	8 (13.3%)	4 (6.8%)	13 (7.3%)	0.05
Cerebrovascular disease n(%)	6 (10%)	13 (21.7%)	10 (16.9%)	29 (16.2%)	0.22
Peripheral vascular disease n(%)	3 (5%)	6 (10%)	8 (13.6%)	17 (9.5%)	0.28
Smoking n(%)	33 (55%)	26 (43.3%)	21 (35.6%)	80 (44.7%)	0.1
Height (in)	67.9 ± 3.5	68 ± 4.1	68 ± 4.1	68 ± 3.9	0.99
Weight (kg)	82.3 ± 28.1	83.9 ± 23.4	80.8 ± 18	82.3 ± 23.5	0.77
Body mass index (kg/m2)	27.7 ± 9.4	28.1 ± 7.2	27.2 ± 6.9	27.7 ± 7.9	0.85
Access type					0.29
Fistula	34 (56.7%)	32 (53.3%)	39 (66.1%)	105 (58.7%)	0.34
Graft	12 (20%)	8 (13.3%)	5 (8.5%)	25 (14%)	0.19
Catheter	14 (23.3%)	20 (33.3%)	15 (25.4%)	49 (27.4%)	0.43
Delivered dialysis duration (min)	220.4 ± 40.5	222.5 ± 36.8	224.4 ± 28.5	222.4 ± 35.5	0.83
Albumin (g/dL)	3.7 ± 0.5	3.6 ± 0.4	3.5 ± 0.4	3.6 ± 0.5	0.19
Hemoglobin (g/dL)	11.6 ± 1.2	11.3 ± 1.5	11.1 ± 1.3	11.3 ± 1.3	0.12

Background antihypertensive treatment of study participants is depicted in Table 2. Almost 96.7% of study participants were previously treated for hypertension, receiving a mean number of 2.7±1.3 drugs daily. Blockers of the renin-angiotensin-aldosterone system and beta-blockers were the most commonly prescribed antihypertensive medications. No difference in the number and nature of BP-lowering drugs was evident among PWV tertiles, with the exception of non-dihydropyridine and dihydropyridine calcium-channel-blockers that were more commonly prescribed in the highest PWV terile.

**Table 2:** Number and class of antihypertensive medications according to the tertile of aortic pulse wave velocity.

Parameter	PWV tertile Low	PWV tertile Medium	PWV tertile High	Overall	P Value
N	60	60	59	179	
Antihypertensive drugs (n)	2.6 ± 1.3	2.7 ± 1.4	2.9 ± 1.2	2.7 ± 1.3	0.42
No antihypertensive drug n(%)	2 (3.3%)	3 (5%)	1 (1.7%)	6 (3.4%)	0.61
ACE inhibitors n(%)	42 (70%)	35 (58.3%)	37 (62.7%)	114 (63.7%)	0.41
ARBs n(%)	5 (8.3%)	6 (10%)	8 (13.6%)	19 (10.6%)	0.64
Beta-blockers n(%)	47 (78.3%)	44 (73.3%)	46 (78%)	137 (76.5%)	0.77
Alpha-blockers n(%)	8 (13.3%)	5 (8.3%)	6 (10.2%)	19 (10.6%)	0.67
Centrally acting agents n(%)	17 (28.3%)	23 (38.3%)	20 (33.9%)	60 (33.5%)	0.51
Non dihydropydine CCBs n(%)	0 (0%)	7 (11.7%)	1 (1.7%)	8 (4.5%)	<0.01
Dihydropydine CCBs n(%)	24 (40%)	27 (45%)	38 (64.4%)	89 (49.7%)	0.02
Vasodilators n(%)	15 (25%)	14 (23.3%)	15 (25.4%)	44 (24.6%)	0.96
Loop diuretics n(%)	0 (0%)	0 (0%)	2 (3.4%)	2 (1.1%)	0.13

**Abbreviations:** PWV = pulse wave velocity; ACE = angiotensin converting enzyme; ARBs = angiotensin receptor blockers; CCBs = calcium channel blockers

### Interdialytic ambulatory BP and indices of left ventricular structure at baseline

As shown in Table 3, higher PWV was associated with elevated mean 44-hour interdialytic ambulatory SBP levels at baseline, whereas mean 44-hour ambulatory DBP and heart rate were no different between PWV tertiles. Baseline levels of LVMI did not significantly differ among PWV subgroups; similarly, increasing baseline aortic PWV was not associated with rise in LVID, IVST and LVPWT.

**Table 3:** Interdialytic ambulatory BP and indices of left ventricular structure at baseline.

Parameter	PWV Tertile Low	PWV Tertile Medium	PWV Tertile High	Overall	P value
N	60	60	59	179	
44-hour ambulatory SBP (mmHg)	148.1 ± 13.4	151.4 ± 13.2	155.8 ± 16.1	151.7 ± 14.5	0.01
44-hour ambulatory DBP (mmHg)	88.8 ± 10.4	88.3 ± 12.7	86.1 ± 12.3	87.8 ± 11.8	0.41
44-hour heart rate (bpm)	79.2 ± 11	82.9 ± 11.7	79.7 ± 13.2	80.6 ± 12	0.19
LVMI (g/m^2^)	178.4 ± 53.6	176.6 ± 51.3	167.7 ± 38.8	174.4 ± 48.5	0.45
IVST (cm)	1.54 ± 0.33	1.58 ± 0.3	1.52 ± 0.25	1.55 ± 0.29	0.54
LVPWT (cm)	1.48 ± 0.3	1.55 ± 0.26	1.47 ± 0.21	1.5 ± 0.26	0.23
LVID (cm)	5.06 ± 0.64	4.95 ± 0.77	4.96 ± 0.63	4.99 ± 0.68	0.64

**Abbreviations:** PWV = pulse wave velocity; SBP = systolic blood pressure; DBP = diastolic blood pressure; LVMI = left ventricular mass index; IVST = interventricular septal thickness; LVPWT = left ventricular posterior wall thickness; LVID = left ventricular internal diameter

### Predictors of LVH at baseline and its longitudinal response to treatment

#### LVMI

**Fig 1:**
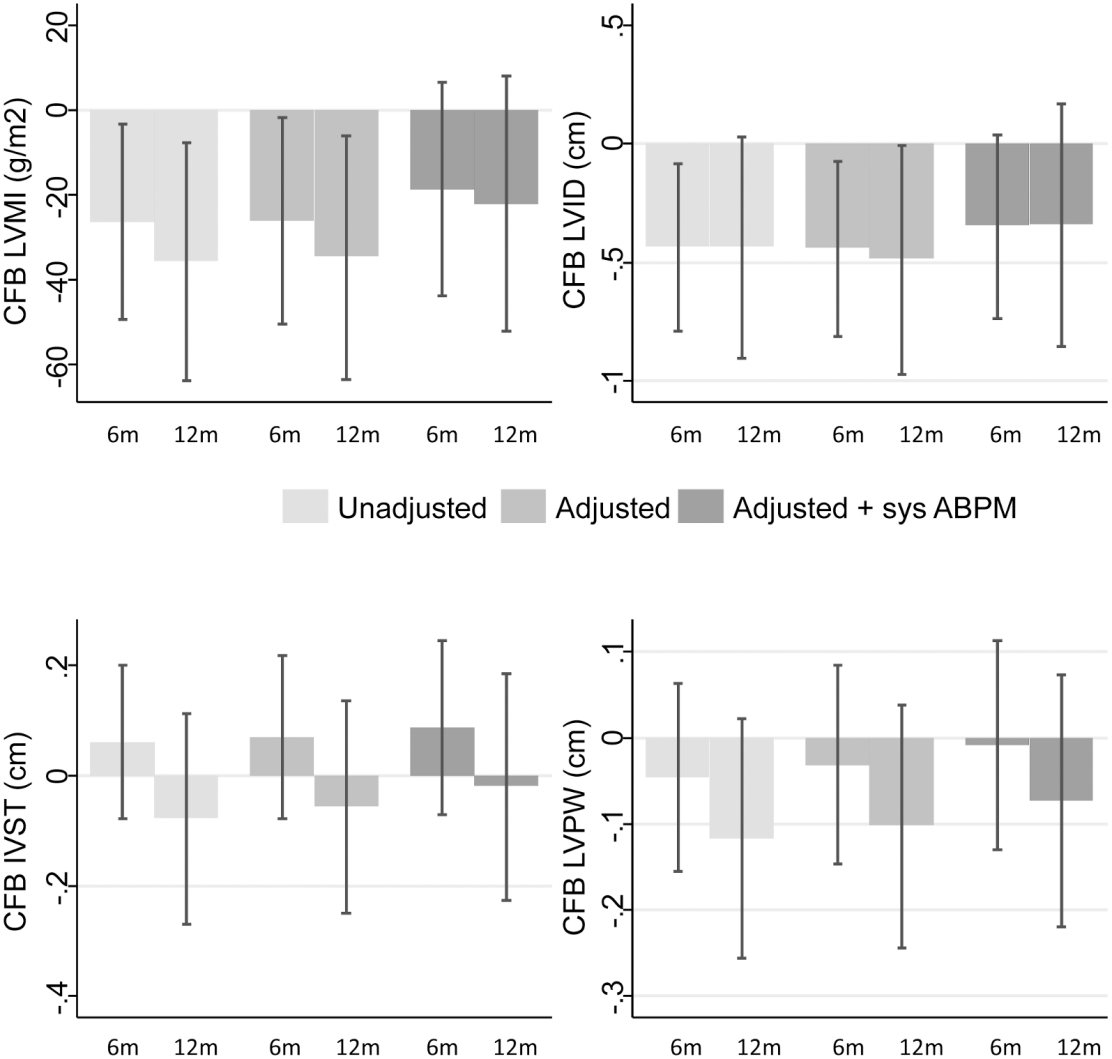
Treatment-induced change in left ventricular mass index and in cavitary and muscular components of left ventricle. The Y-axis shows the change from baseline (CFB) to 6 and 12 months of treatment in a) left ventricular mass index (LVMI); b) left ventricular internal diameter (LVID); c) interventricular septal thickness (IVST); and d) left ventricular posterior wall thickness (LVPWT). The x-axis shows the months of treatment. Shades of the bars represent progressive adjustments to the models with the darker bar representing the full-adjusted models. The error bars represent 95% confidence intervals of the means derived from mixed linear model analysis. In unadjusted analysis, LVMI and LVID were significantly reduced from baseline to 6 and 12 months in contrast to IVST and LVPWT that remained unchanged. After adjustment for age, gender, race, smoking, diabetic status, history of previous cardiovascular disease, hemoglobin levels, treatment arm and drug effect over time, CFB to 6 and 12 months in LVMI and LVID remained significant; however, additional adjustment for the CFB in 44-hour ambulatory systolic blood pressure or IVC diameter mitigated the reduction in LVMI and LVID. IVST and LVPWT remained constant during follow-up in both adjusted models.

Table 4 and Fig 1 show the predictors of LVMI change over time. In unadjusted analysis, significant effect of visits indicates that LVMI was significantly reduced from baseline to 6 months (β -26.2 g/m^2^; p = 0.03) and from baseline to 12 months (β -35.7 g/m^2^ p = 0.01). The interaction term of PWV x visit was not significant indicating no association of PWV with regression of LVMI. The aortic PWV was neither associated with LVMI at baseline (β -1.6 g/m^2^ p = 0.23) nor with LVMI decline during follow-up (overall p value for PWV*visit interaction term = 0.16) (Table 4).

**Table 4:** Progressively adjusted models of change from baseline in left ventricular mass index.

Model	Mean (95% CI)	P value	Overall P value
**Model 1: Unadjusted**			
6-month visit	-26.2(-49.2–-3.3)	0.03	0.02
12-month visit	-35.7 (-63.7–-7.6)	0.01	
6-month visit*PWV	2.7 (-0.2–5.5)	0.07	0.16
12-month visit*PWV	2.1 (-1.5–5.7)	0.26	
PWV at baseline visit	-1.6 (-4.2–1.0)	0.23	
Constant	186.5 (165.4–207.5)	<0.001	
**Model 2: Adjusted for age, sex, race, smoking, diabetes, CVD, treatment arm, drug effect over time**			
6-month visit	-26.0 (-50.2–-1.9)	0.03	0.02
12-month visit	-34.8 (-63.4–-6.1)	0.02	
6-month visit*PWV	2.4 (-0.4–5.3)	0.09	0.22
12-month visit*PWV	1.9 (-1.7–5.5)	0.3	
PWV at baseline visit	-2.0 (-4.7–0.7)	0.15	
Constant	254.4 (186.6–322.2)	<0.001	
**Model 3: adjusted for factors in model 2 +ambulatory SBP at baseline and over time**			
6-month visit	-18.6 (-43.7–6.5)	0.15	0.22
12-month visit	-22.1 (-52.2–8.0)	0.15	
6-month visit*PWV	2.7 (-0.2–5.7)	0.07	0.19
12-month visit*PWV	1.5 (-2.2–5.2)	0.43	
PWV at baseline visit	-2.3 (-5.1–0.4)	0.09	
Constant	203.5 (105.1–301.9)	<0.001	
**Model 4: adjusted for factors in model 2 +IVC diameter at baseline and over time**			
6-month visit	-6.6 (-41.6–28.4)	0.71	0.91
12-month visit	0.6 (-39.5–40.7)	0.98	
6-month visit*PWV	2.0 (-0.9–4.8)	0.18	0.41
12-month visit*PWV	0.9 (-2.8–4.6)	0.64	
PWV at baseline visit	-1.7 (-4.4–1.0)	0.23	
Constant	224.9 (152.9–297.0)	<0.001	

**Abbreviations:** CI = confidence intervals of the mean; PWV = pulse wave velocity; CVD = cardiovascular disease; SBP = systolic blood pressure; IVC = inferior vena cava diameter (cm/m^2^) in expiration. Change from baseline in LVMI is expressed in g/m^2^. The PWV*visit interaction term is expressed as g/m^2^ change in LVMI per each 1 m/sec increase in PWV.

After adjustment for age, gender, race, smoking, diabetic status, history of previous cardiovascular disease, hemoglobin levels, treatment arm and drug effect over time, decrease in LVMI from baseline to 6 months (β -26.0, p = 0.03) and baseline to 12 months (β -34.8, p = 0.02) remained statistically significant.

Additional adjustment (of Model 2) for the change in ambulatory SBP over time (Model 3 in Table 4) mitigated the treatment-induced reduction in LVMI (β -18.6, p = 0.15 at 6-months and β -22.1, p = 0.15 at 12-months) (see also Fig 1). Again, aortic PWV was neither an independent determinant of LVMI at baseline (p = 0.09) nor a predictor of the change in LVMI during follow-up (overall p value for the PWV*visit interaction term = 0.19).

To further explore the provenance of LVMI reduction, additional adjustment (of Model 2) for the change in IVC diameter over time (Model 4 in Table 4) essentially abolished the treatment-induced reduction in LVMI.

#### Cavitary and muscular components of left ventricle

As shown in Fig 1, in unadjusted analysis, significant treatment-induced reductions from baseline to 6 and 12 months were evident for LVID (β -0.44 for the 6-month visit and β -0.44 for the 12-month visit, p = 0.03 for overall visit effect). In contrast, treatment with either lisinopril- or atenolol-based regimen was not associated with improvement in the LV muscular compartment, as both IVST and LVPWT remained unchanged during the 12-month follow-up period (Fig 1).

After adjustment for several cardiovascular risk factors (age, gender, black race, smoking status, presence of diabetes, history of pre-existing cardiovascular disease, hemoglobin, randomized drug and drug effect over time), change in LVID from baseline to 6 months (β -0.44; 95% CI: -0.81, -0.07, p = 0.02) as well as from baseline to 12 months (β -0.49; 95% CI: -0.97, -0.01, p = 0.047) remained significant (overall visit effect p = 0.03). Additional adjustment for ambulatory SBP at baseline and its change throughout the trial removed the effect of treatment on the change in LVID at 6 months (β -0.35; 95% CI: -0.73, 0.04, p = 0.08) and 12 months of follow-up (β -0.34; 95% CI: -0.85, 0.17, p = 0.19). Similarly, adjustment for IVC diameter at baseline and its change over time removed the effect of treatment-induced change in LVID at any time point. The overall visit effect became non-significant (p = 0.17). As shown in Fig 1, both IVST and LVPWT remained unchanged over time in adjusted analyses.

## Discussion

Major findings of the present study were that among hypertensive hemodialysis patients with echocardiographic LVH: (i) increasing aortic PWV was not determinant of the LVMI at baseline and was unable to predict the treatment-induced reduction in LVMI over 12 months of follow-up; (ii) treatment-induced decline in LVMI was independent from age, gender, race, several other cardiovascular risk factors, treatment arm and drug effect over time; (iii) treatment-induced decline in LVMI was mitigated by reduction in ambulatory SBP which supports the hypothesis that volume is in the causal pathway of decline in LVMI; (iv) treatment-induced decline in LVMI was mitigated by reduction in IVC diameter which directly supports the notion that volume is in the causal pathway of decline in LVMI; (v) lowering of LVMI during the 12-month follow-up was mediated predominantly through reduction in the cavitary component of left ventricle instead of improvement in LV wall thickness. Taken together, these observations support the notion that volume (rather than arterial stiffness) is major mediator of decline in LVMI.

The finding of the present study that aortic PWV was neither determinant of baseline LVMI nor predictor of the treatment-induced decline in LVMI over time is in line with the results of previous studies conducted in the general hypertensive population, showing that impaired mechanical properties of large arteries did not influence the efficacy of antihypertensive treatment in causing regression of LVH [16, 17]. In an observational analysis of 61 patients with essential hypertension, Hashimoto et al. [16] showed that neither aortic PWV nor transit time of pulse wave were predictors of reduction in LVMI after 12 months of antihypertensive treatment. This is strongly supported by the results of a post-hoc analysis of the Losartan Intervention For Endpoint reduction in hypertension (LIFE) echo sub-study [17], in which reduced ratio of pulse pressure to stroke volume (PP/SVi), an index reflective of systemic arterial compliance, was associated with greater BP reduction, but was unable to predict the decline in LVMI over 5-year-long treatment with either losartan or atenolol in 960 patients with essential hypertension and echocardiographic LVH.

The most important finding of the present work was that LVMI lowering in the HDPAL trial was strongly associated with the change in 44-hour ambulatory SBP (a proxy of achievement of dry weight) and with change in IVC diameter (a proxy for achievement of volume control) during follow-up. Indeed, additional adjustment of the models for the longitudinal reduction in interdialytic BP load or IVC diameter removed the significant effect of visits on LVMI and LVID, indicating that LVH regression was largely volume-mediated (Fig 1). In addition, among cavitary and muscular compartments of left ventricle, the present study showed that treatment-induced regression of LVMI was predominantly due to reduction in LV chamber dilatation rather than improvement in LV wall thickness. It is likely that such an effect (i.e., decrease in LVID but not in IVST and LVPWT) may be due to better achievement of dry weight over time [18, 19]. These findings are in sharp contrast to those reported in populations without ESRD such as in the LIFE study which used non-volume reduction strategies to reduce BP [20]. In the LIFE study, treatment-induced reduction in LVMI with either atenolol or losartan was predominantly due to reductions in thickness of the ventricular wall (IVST and LVPWT). In fact, with either drug, the LVID increased [20]. Diuretic use was no different in the two treatment groups. In the Trial of Mild Hypertension Study, treatment-induced reduction in LVMI over one year was reported for 5 antihypertensive agents: amlodipine, acebutalol, doxazosin, enalapril and chlorthalidone [21]. Compared to others, greatest reduction in LVID was seen with chlorthalidone treatment. Together these findings suggest that among hypertensive hemodialysis patients with echocardiographic LVH, strict volume control may be the predominant mechanism that evokes a reduction in LVMI. This study was conducted in a subset of dialysis patients treated with specific antihypertensive medications within a randomized controlled trial. However, volume-mediated improvement in LVMI may be relevant for the whole hemodialysis population, since probing of dry-weight represents the main non-pharmaceutical therapeutic approach to achieve adequate BP control in these patients [22]. These findings are also consistent with non-ESRD populations where a volume reduction strategy such as with diuretics also evokes reduction in LVMI.

In the Dry Weight Reduction in Hypertensive Hemodialysis Patients (DRIP) trial, we have previously shown that among 150 hypertensive hemodialysis patients, probing of dry weight over a 24-dialysis treatment period culminated in a significantly greater reduction of 10.9 g/m2 in LVMI as compared to the standard ultrafiltration [6]. The present study expands our previous observations, showing that over a longer 12-month treatment period reduced exposure of myocardium to the interdialytic volume and pressure overload was the major pathway of LVH regression. This notion is also supported by another recent study, in which volume management through dietary sodium restriction and intensified ultrafiltration during dialysis was associated with reduced LVMI and improved LV function at both systole and diastole [23]. In a recent pilot study, strict volume control through bioelectrical impendence-guided management of dry-weight was associated with greater improvement in LVMI in comparison with the clinically-guided adjudication of volume status [24]. In addition, the Frequent Hemodialysis Network (FHN) trial showed that short daily hemodialysis, a treatment intervention that enhances volume withdrawal over a longer dialysis time and probes achievement of dry weight, caused a greater regression in LVMI relative to conventional thrice-weekly hemodialysis over 12 months of follow-up [25].

This study has several strengths and limitations. Important strength of this work was that the protocol prespecified echocardiographic evaluation of LV structure at repeated time-points during follow-up to adequately capture treatment-induced changes in LVMI and related parameters in the context of a randomized controlled trial. To mitigate the effect of volume on cavity dimensions, we fixed the time of echocardiography to immediately post dialysis. Strength of this study was also the use of the most reliable method of 44-hour ABPM in an attempt to properly evaluate the true BP load imposed to the left ventricle during the entire interdialytic period. Of note, peridialytic BP recordings exhibit extensive variability, poor reproducibility and provide inaccurate estimates of 44-hour interdialytic ambulatory BP [22, 26]. Furthermore, we used a direct and validated measure of volume—echocardigraphically measured IVC diameter. However, this study has also some limitations. This study did not include determination of central aortic pressures and wave reflection indices, which are also important components arterial cushioning function [7]; thus, whether amplitude and timing of wave reflections from peripheral vascular beds are determinants of LVMI at baseline or predictors of its longitudinal change cannot be established in the present study. Further, echo-derived measurements of aortic PWV were not previously validated against invasive (gold-standard) measurements in dialysis patients; however, none of the currently available techniques for non-invasive aortic PWV assessment was particularly tested against intra-aortic measurements in this specific population. Last, his study is an analysis of a single-center trial and a large number of participants were African-Americans; whether the results of this study are generalizable in other patient populations with different racial and ethnic characteristics remains uncertain.

## Conclusion

Among hypertensive hemodialysis patients with echocardiographic LVH, volume-mediated reduction in interdialytic ambulatory BP but not severity of aortic stiffness is a more important predictor of treatment-induced reduction in LVMI. To the extent this cause-and-effect association is true, strict volume control and probing of dry weight appear as effective therapeutic approaches to improve LVH and potentially reduce risk of cardiovascular morbidity and mortality in hemodialysis patients.

## Supporting Information

S1 Data (ZIP)

## References

[1] Levy D. Clinical significance of left ventricular hypertrophy: insights from the Framingham Study. *J Cardiovasc Pharmacol* 1991;17 Suppl 2:S1–S6. 10.1097/00005344-199117002-00002 1715449

[2] Levin A, Thompson CR, Ethier J, Carlisle EJ, Tobe S, Mendelssohn D, et al Left ventricular mass index increase in early renal disease: impact of decline in hemoglobin. *Am J Kidney Dis* 1999 7;34(1):125–34. 10401026 10.1016/s0272-6386(99)70118-6

[3] Zoccali C, Benedetto FA, Mallamaci F et al Prognostic impact of the indexation of left ventricular mass in patients undergoing dialysis. *J Am Soc Nephrol* 2001 12;12(12):2768–74. 11729247 10.1681/ASN.V12122768

[4] Zoccali C, Benedetto FA, Mallamaci F, Tripepi G, Giacone G, Cataliotti A, et al Left ventricular mass monitoring in the follow-up of dialysis patients: prognostic value of left ventricular hypertrophy progression. *Kidney Int* 2004 4;65(4):1492–8. 15086493 10.1111/j.1523-1755.2004.00530.x

[5] Bossola M, Tazza L, Vulpio C, Luciani G. Is regression of left ventricular hypertrophy in maintenance hemodialysis patients possible? *Semin Dial* 2008 9;21(5):422–30. 18764802 10.1111/j.1525-139X.2008.00471.x

[6] Agarwal R, Bouldin JM, Light RP, Garg A. Probing dry-weight improves left ventricular mass index. *Am J Nephrol* 2011;33(4):373–80. 21447945 10.1159/000326235 PMC3078237

[7] Laurent S, Cockcroft J, Van Bortel L, Boutouyrie P, Giannattasio C, Hayoz D, et al Expert consensus document on arterial stiffness: methodological issues and clinical applications. *Eur Heart J* 2006 11;27(21):2588–605. 17000623 10.1093/eurheartj/ehl254

[8] London G, Covic A, Goldsmith D, Wiecek A, Suleymanlar G, Ortiz A, et al Arterial aging and arterial disease: interplay between central hemodynamics, cardiac work, and organ flow-implications for CKD and cardiovascular disease. *Kidney Int Suppl (2011)* 2011 6;1(1):10–2. 25018896 10.1038/kisup.2011.5 PMC4089718

[9] London GM, Guerin AP, Marchais SJ, Pannier B, Safar ME, Day M, et al Cardiac and arterial interactions in end-stage renal disease. *Kidney Int* 1996 8;50(2):600–8. 8840292 10.1038/ki.1996.355

[10] Nitta K, Akiba T, Uchida K, Otsubo S, Otsubo Y, Takei T, et al Left ventricular hypertrophy is associated with arterial stiffness and vascular calcification in hemodialysis patients. *Hypertens Res* 2004 1;27(1):47–52. 15055255 10.1291/hypres.27.47

[11] Agarwal R, Sinha AD, Pappas MK, Abraham TN, Tegegne GG. Hypertension in hemodialysis patients treated with atenolol or lisinopril: a randomized controlled trial. *Nephrol Dial Transplant* 2014 3;29(3):672–81. 24398888 10.1093/ndt/gft515 PMC3938300

[12] Devereux RB, Alonso DR, Lutas EM, Gottlieb GJ, Campo E, Sachs I, et al Echocardiographic assessment of left ventricular hypertrophy: comparison to necropsy findings. *Am J Cardiol* 1986 2 15;57(6):450–8. 2936235 10.1016/0002-9149(86)90771-x

[13] Agarwal R, Bouldin JM, Light RP, Garg A. Inferior vena cava diameter and left atrial diameter measure volume but not dry weight. *Clin J Am Soc Nephrol* 2011 2 17;6(5):1066–72. 21330484 10.2215/CJN.09321010 PMC3087772

[14] Lehmann ED, Riley WA, Clarkson P, Gosling RG. Non-invasive assessment of cardiovascular disease in diabetes mellitus. *Lancet* 1997 7;350 Suppl 1:SI14–SI19. 9250278 10.1016/s0140-6736(97)90023-4

[15] Parati G, Stergiou G, O'Brien E, Asmar R, Beilin L, Bilo G, et al European Society of Hypertension practice guidelines for ambulatory blood pressure monitoring. *J Hypertens* 2014 7;32(7):1359–66. 24886823 10.1097/HJH.0000000000000221

[16] Hashimoto J, Westerhof BE, Westerhof N, Imai Y, O'Rourke MF. Different role of wave reflection magnitude and timing on left ventricular mass reduction during antihypertensive treatment. *J Hypertens* 2008 5;26(5):1017–24. 18398345 10.1097/HJH.0b013e3282f62a9b

[17] Palmieri V, Bella JN, Gerdts E, Wachtell K, Papademetriou V, Nieminen MS, et al Change in pulse pressure/stroke index in response to sustained blood pressure reduction and its impact on left ventricular mass and geometry changes: the life study. *Am J Hypertens* 2008 6;21(6):701–7. 18437127 10.1038/ajh.2008.162

[18] Drighil A, Madias JE, Mathewson JW, El Mosalami H, El Badaoui N, Ramdani B, et al Haemodialysis: effects of acute decrease in preload on tissue Doppler imaging indices of systolic and diastolic function of the left and right ventricles. *Eur J Echocardiogr* 2008 7;9(4):530–5. 18490307 10.1093/ejechocard/jen125

[19] Zoccali C. Left ventricular mass index as an outcome measure in clinical trials in dialysis patients: a word of caution. *Am J Nephrol* 2011;33(4):370–2. 21447944 10.1159/000326239

[20] Devereux RB, Dahlof B, Gerdts E, Boman K, Nieminen MS, Papademetriou V, et al Regression of hypertensive left ventricular hypertrophy by losartan compared with atenolol: the Losartan Intervention for Endpoint Reduction in Hypertension (LIFE) trial. *Circulation* 2004 9 14;110(11):1456–62. 15326072 10.1161/01.CIR.0000141573.44737.5A

[21] Liebson PR, Grandits GA, Dianzumba S, Prineas RJ, Grimm RH Jr, Neaton JD, et al Comparison of five antihypertensive monotherapies and placebo for change in left ventricular mass in patients receiving nutritional-hygienic therapy in the Treatment of Mild Hypertension Study (TOMHS). *Circulation* 1995 2 1;91(3):698–706. 7828296 10.1161/01.cir.91.3.698

[22] Agarwal R, Flynn J, Pogue V, Rahman M, Reisin E, Weir MR. Assessment and Management of Hypertension in Patients on Dialysis. *J Am Soc Nephrol* 2014 4 3. 10.1681/ASN.2013060601 PMC4116052 24700870

[23] Kayikcioglu M, Tumuklu M, Ozkahya M, Ozdogan O, Asci G, Duman S, et al The benefit of salt restriction in the treatment of end-stage renal disease by haemodialysis. *Nephrol Dial Transplant* 2009 3;24(3):956–62. 19004849 10.1093/ndt/gfn599

[24] Hur E, Usta M, Toz H, Asci G, Wabel P, Kahvecioglu S, et al Effect of fluid management guided by bioimpedance spectroscopy on cardiovascular parameters in hemodialysis patients: a randomized controlled trial. *Am J Kidney Dis* 2013 6;61(6):957–65. 23415416 10.1053/j.ajkd.2012.12.017

[25] Chertow GM, Levin NW, Beck GJ, Depner TA, Eggers PW, Gassman JJ, et al In-center hemodialysis six times per week versus three times per week. *N Engl J Med* 2010 12 9;363(24):2287–300. 21091062 10.1056/NEJMoa1001593 PMC3042140

[26] Sinha AD, Agarwal R. Peridialytic, intradialytic, and interdialytic blood pressure measurement in hemodialysis patients. *Am J Kidney Dis* 2009 11;54(5):788–91. 19853196 10.1053/j.ajkd.2009.07.004 PMC2784813

